# Innate Immune Responses of Pulmonary Epithelial Cells to *Burkholderia pseudomallei* Infection

**DOI:** 10.1371/journal.pone.0007308

**Published:** 2009-10-06

**Authors:** Siew Hoon Sim, Yichun Liu, Dongling Wang, Vidhya Novem, Suppiah Paramalingam Sivalingam, Tuck Weng Thong, Eng Eong Ooi, Gladys Tan

**Affiliations:** 1 Defence Medical and Environmental Research Institute, DSO National Laboratories, Singapore, Republic of Singapore; 2 Program in Emerging Infectious Diseases, Duke-NUS Graduate Medical School, Singapore, Republic of Singapore; Singapore Immunology Network, Singapore

## Abstract

**Background:**

*Burkholderia pseudomallei*, a facultative intracellular pathogen, causes systemic infection in humans with high mortality especially when infection occurs through an infectious aerosol. Previous studies indicated that the epithelial cells in the lung are an active participant in host immunity. In this study, we aimed to investigate the innate immune responses of lung epithelial cells against *B. pseudomallei*.

**Methodology and Principal Findings:**

Using a murine lung epithelial cell line, primary lung epithelial cells and an inhalational murine infection model, we characterized the types of innate immunity proteins and peptides produced upon *B. pseudomallei* infection. Among a wide panel of immune components studied, increased levels of major pro-inflammatory cytokines IL-6 and TNFα, chemokine MCP-1, and up-regulation of secretory leukocyte protease inhibitor (SLPI) and chemokine (C-C motif) ligand 20 (CCL20) were observed. Inhibition assays using specific inhibitors suggested that NF-κB and p38 MAPK pathways were responsible for these *B. pseudomallei-*induced antimicrobial peptides.

**Conclusions:**

Our findings indicate that the respiratory epithelial cells, which form the majority of the cells lining the epithelial tract and the lung, have important roles in the innate immune response against *B. pseudomallei* infection.

## Introduction

*Burkholderia pseudomallei*, the causative agent for melioidosis, is an environmental saphrophyte that is found most commonly in Southeast Asia and Northern Australia [1]–[3]. In endemic regions such as northeast Thailand, infections by *B. pseudomallei* account for up to 36% of fatal community-acquired pneumonia [4]. Infection results in a diverse array of clinical manifestations ranging from acute septicemia to chronic disease, and can occur via multiple routes such as ingestion, inhalation or percutaneous inoculation [5]. The inhalational route of infection is associated with high mortality rates [6], [7].

Epidemiological observations have indicated that onset of pulmonary infections can develop as early as 2 days following an inhalational exposure of *B. pseudomallei*, especially for individuals with pre-existing medical conditions such as diabetes mellitus and renal failure [6], [8]. A study in Australia indicated a significant increased incidence of melioidosis cases with pneumonia and septicaemia during the monsoon season with heavy rainfall [6]. In Banda Aceh, a month after the Asian tsunami, *B. pseudomallei* was cultured from the pleural fluids of 25 patients [9]. In Singapore, a significantly high percentage of pneumonic melioidosis cases (76.7% of 43 cases) was observed during a three month monsoon season in early 2004 compared to the annual mean (from 1999 to 2003) of 33.9% [10]. It is postulated that infectious aerosol generated with the monsoon rain lead to increased incidence of pneumonic melioidosis.

Unlike other internal organs, the vulnerability of the lungs to infection arises from the continuous exposure of its vast surface area (150 m^2^) to a large variety and quantity of microorganisms in the inhaled air [11], and hence are vulnerable to inhalational inoculation with *B. pseudomallei*. Furthermore, the rich network of capillaries also makes it vulnerable to hematogenous spread of *B. pseudomallei*.

In *B. pseudomallei* infection, macrophages are well-known to be the sentinel cells involved in host immune defense against the bacteria. Upon activation, alveolar macrophages can initiate a series of inflammatory responses that are targeted at clearing the inoculum [12]–[14]. Recent evidence from studies on other lung pathogens, however, suggests that pulmonary epithelial cells, which are among the first cells to come into contact with the pathogen, also play an important role in host innate immunity [15]–[17]. Infection of human lung epithelial cells with *Mycoplasma pneumoniae* resulted in increased expression of proinflammatory cytokines interleukin IL-8, tumour necrosis factor alpha (TNFα) and IL-1β mRNA [17]. *Mycobacterium bovis* bacillus Calmette-Guérin (BCG) was also able to stimulate antimicrobial peptide, human beta-defensin-2 (HBD-2) mRNA expression in human lung epithelial cells [15]. Hence, the extensive studies on *B. pseudomallei* pathogenesis using monocyte/macrophages may have missed the important interaction between the bacterium and the lung epithelial cells. Although an earlier study tried to address the response of lung epithelial cells to *B. pseudomallei* infection, it focused only on IL-8 production [18]. In this study, we utilized a murine lung epithelial cell line, primary murine lung epithelial cells as well as a murine model of inhalational infection to elucidate in greater detail the role of lung epithelial cells in the innate immune response to *B. pseudomallei*. A wide spectrum of important immune proteins and antimicrobial peptides in lung epithelial cells were examined. Our data suggests that the epithelium which constitutes the majority of cells in the lung is capable of eliciting a wide spectrum of key immune proteins and peptides against *B. pseudomallei*, indicating that epithelial cells, like macrophages, are also sentinel cells in the lung and these cells are important for first line of defence against inhaled *B. pseudomallei*.

## Methods

### Animals

Female BALB/c and C57Bl/6 mice aged 6- to 8-week old were purchased from the Center for Animal Resources in National University of Singapore. The mice were maintained in polystyrene cages with shaved wood bedding and fed with commercial pellets (PMI Nutrition International, USA) prior to the experiment. All animal procedures were approved (in writing) by the Animal Care and Use Committee, DSO National Laboratories.

### Bacterial strain and culture

*B. pseudomallei*, KHW, was obtained from the *B. pseudomallei* strains collection at DMERI, DSO National Laboratories. For infection assays, single colony of KHW was grown overnight in 5 ml tryptone soy broth (TSB) (Difco Laboratories, Detroit, Michigan) at 37°C for 16 h. The bacterial culture was then diluted 1∶20 in 20 ml TSB and grown for 3 h at 37°C for the bacteria to reach log phase. Subsequently, the bacteria pellet was harvested by centrifugation at 2000 g for 15 min and resuspended in Kaighn's modification of Ham's F12 (F12K) medium to an OD_600 nm_ of 1.0 (equivalent to a concentration of 10^8^ bacteria per ml) and used for subsequent infection assays.

### Cell line and culture

All cell culture reagents were purchased from Gibco BRL (Grand Island, N.Y.) unless otherwise stated. Murine lung epithelial cell line, LA-4, was purchased from American Type Culture Collection (ATCC) (Manassas, VA) and grown in F12K medium containing 15% heat-inactivated fetal bovine serum at 37°C in 5% CO_2_.

### Preparation of primary lung epithelial cells from BALB/c and C57Bl/6 mice

**Figure 1:**
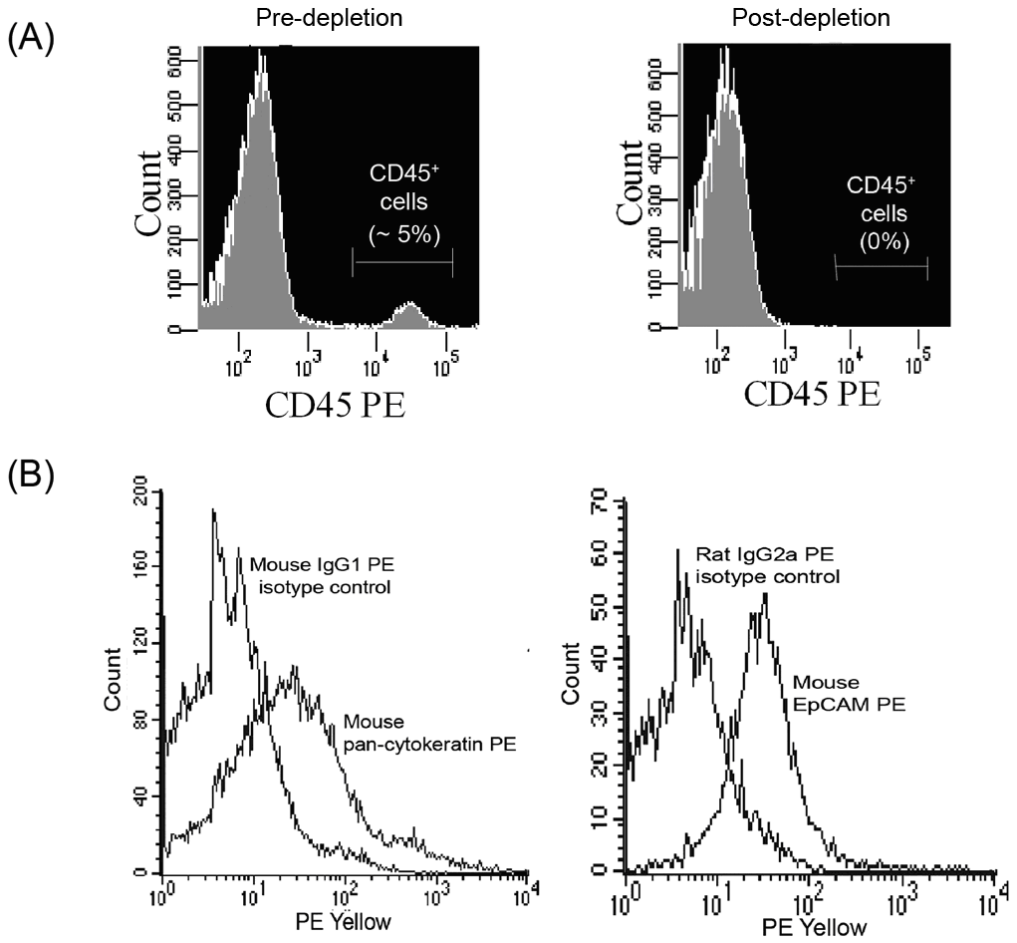
Analysis of the purified primary murine lung epithelial cells by flow cytometry analysis using FACSArray. The epithelial cells were harvested using a series of centrifugation steps followed by depletion of CD45^+^ cells. The absence of CD45^+^ cells from the primary lung epithelial cell preparation was confirmed by cell surface staining using antibody against CD45^+^ cells (A). The presence of the epithelial lineage in the primary lung cell preparation isolated from BALB/c mice was confirmed by cell surface staining using antibodies against pan-cytokeratin and EpCAM (B).

Isolation of primary epithelial cells from the lungs of BALB/c and C57Bl/6 mice was performed as previously described [19], with slight modifications. Briefly, lungs were excised from 15 BALB/c or C57Bl/6 mice and cut into 1-mm^3^ pieces. Epithelial cells were then dissociated from the lung tissues with collagenase A (2 mg/ml) (Roche Diagnostics GmBH, Mannheim, Germany) in the presence of penicillin (100 IU/ml)-streptomycin (100 µg/ml) (Sigma-Aldrich, St Louis, MO) in F12K medium for 2 h at 37°C. The cells were then centrifuged at 1000 g for 10 min and treated with F12k containing 2 U/ml Deoxyribonuclease I (Invitrogen, Carlsbad, California) for 5 min at 25°C. The epithelial organoids were then harvested at 500 g for 15 s. This step was repeated six times. Subsequently, the pellet of epithelial organoids was then treated with red blood cell (RBC) lysis buffer for 10 min at 25°C and washed once with F12K medium. The cells were stained with anti-mouse CD45 PE (BD Biosciences, San Diego, CA), and depletion of CD45^+^ cells was carried out using anti-PE microbeads (Miltenyi-Biotech, California, USA). After the purification procedure, the absence of CD45^+^ cells was verified by flow cytometry analysis with anti-mouse CD45 PE. No CD45^+^ cells were detected (Figure 1A). The epithelial lineage of 20000 isolated cells was confirmed by staining with anti-mouse pan-cytokeratin PE and anti-mouse epithelial cell adhesion molecule (EpCAM) PE (Santa Cruz Biotechnology, California, USA). FACS analysis was performed with FACSArray and CellQuest software (BD Biosciences, San Diego, CA) and isotype antibodies were used as controls to set the gate. All isolated cells from BALB/c and C57Bl/6 mice stained positive for mouse pan-cytokeratin and EpCAM, confirming their epithelial lineage. A representative histogram plot for BALB/c mice was shown in Figure 1B. The viability of the isolated cells was checked by Trypan-Blue exclusion and found to be greater than 96%. All isolated epithelial cells were maintained in F12K medium supplemented with 10% heat-inactivated fetal bovine serum at 37°C in 5% CO_2_ for 2 days prior to use.

### Invasion assays

LA-4 cells were seeded at a density of 2.5×10^6^ cells/ml into 6-well plates (Nunc, Denmark) and primary lung epithelial cells were seeded at a density of 2×10^5^ cell /ml in 96-well plate (Nunc, Denmark). Invasion assay was performed using a method described previously by Elsinghorst (1994) [20] with slight modifications. The cultured lung epithelial cells were infected with KHW at a multiplicity of infection of 1∶5 for 2 h. For the negative control, F12K medium was added instead of live bacteria. Subsequently, the cells were washed thrice with 1X PBS and covered with F12K medium containing 250 µg/ml kanamycin to eliminate the extracellular bacteria. After the bacterial infection described above, the supernatant at 0 h, 2 h, 6 h, 24 h and 48 h post infection was collected and filtered through a 0.22 µm filter (Millipore Corporation, Billerica, MA) for further cytokine analysis.

To determine the intracellular bacteria, the cells were washed thrice with 1X PBS and lysed with 1% triton X-100 (Sigma-Aldrich, St Louis, MO). Viable intracellular bacteria were counted by plating onto TSA agar plates. The assay was repeated twice and each assay was carried out in triplicates, results were expressed as log_10_ of CFU per 10^6^ LA-4 cells.

For NF-κB and p38 MAPK pathways inhibition experiments, LA-4 cells were pre-incubated with or without 10 µM of BAY11-7082 and SB203580 (Calbiochem, San Diego, California) for 1 h prior to the infection process.

### Cytokine assays

The presence of IL-10, IL-12p70, IFNγ, IL-6, MCP-1 and TNFα in the supernatants was analyzed using FACSArray Bioanalyzer and Cytometric Bead Array according to the manufacturer's instructions (BD Biosciences, San Diego, CA). Briefly, culture supernatant was incubated with equal volume of each cytokine-specific capture beads for 1 h at room temperature. The detection reagent was then added to the reaction mixture for 1 h prior to analysis on FACSArray Bioanalyzer. The assay was repeated twice and each assay was carried out in triplicates.

### RNA isolation and quantitive real-time RT-PCR (qRT-PCR)

Total RNA was isolated from the epithelial cells using TRizol (Invitrogen, Carlsbad, California). At different time-points post infection, the medium was completely removed and TRizol was added to epithelial cells for 5 min. Following that, 0.2 ml chloroform per ml of TRizol was added and the mixture was centrifuged at 10000 g for 30 min at 4°C. The aqueous layer was collected and RNA was precipitated with isopropanol.

The RNA was then reverse transcribed to cDNA using the SuperScript III platinum two-step qRT-PCR kit (Invitrogen, Carlsbad, California). Quantitative PCR (qPCR) on iCycler iQ (Biorad) was carried out as follows: single UDG incubation step at 50°C for 2 min, single pre-denaturation step at 95°C for 2 min, 45 cycles of 15 s at 95°C and 1 min at 60°C. The sequences of the primers were given in Table 1. The fold change in the sample gene, given by delta-delta C_t_ (ΔΔC_t_), is determined by the following equation: ΔΔC_T_  =  ΔC_T_ (sample)−ΔC_T_ (calibrator), and ΔC_T_ is the C_T_ of the target gene (sample or calibrator) subtracted from the C_t_ the housekeeping gene, actin. [21]. Using this method, a positive fold change would represent up-regulation of the sample gene compared to the calibrator; whereas a negative fold change would represent down-regulation of the sample gene compared to the calibrator.

**Table 1:** Sequences of primers and probes for qRT-PCR.

Gene		Primer sequence
Lysozyme	forward	5′**-**AACCCCAAGAGCTGTGAATG-3’
	Reverse	5′-GGACAGATCTCGGTTTTGA-3′
	Probe	5′-TGCCACCCATGCTCGAATGC-3′
Secretory Leukocyte protease inhibitor (SLPI)	forward	5′-GTTCCAAGTGCGTGAATCCT-3′
	Reverse	5′-GTCAGGGATCAGGCTCACAT-3′
	Probe	5′-TTGCCGTCACACTGCCCGTC-3′
Chemokine (C-C motif) ligand 20 (CCL20)	forward	5′-CGTCTGCTCTTCCTTGCTTT-3′
	Reverse	5′-CTTCATCGGCCATCTGTCTT-3′
	Probe	5′-TGCTTGCTTCTGCCTGGCTGC-3′
β-Actin	forward	5′-AGAGGGAAATCGTGCGTGAC-3′
	Reverse	5′-CAATAGTGATGACCTGGCCGT-3′
	Probe	5′-CACTGCCGCATCCTCTTCCTCCC-3′
Toll-like receptor 2 (TLR2)	forward	5′-GCGGACTGTTTCCTTCTGAC-3′
	Reverse	5′-CCAAAGAGCTCGTAGCATCC-3′
	Probe	5′-GCATCCGAATTGCATCACCGG-3′
Toll-like receptor 4 (TLR4)	forward	5′-CAGCAAAGTCCCTGATGACA-3′
	Reverse	5′-AGAGGTGGTGTAAGCCATGC-3′
	Probe	5′-TCACACCTGGATAAATCCAGCCACTG-3′
Murine cathelicidin-related antimicrobial peptide (CRAMP)	forward	5′-TGGATGACTTCAACCAGCAG-3′
	reverse	5′-TCCTTCACTCGGAACCTCAC-3′
	Probe	5′-TCCTCGTCCCCTTGGGGCTC-3′
Surfactant protein A	forward	5′-AAGGGAGAGCCTGGAGAAAG-3′
	reverse	5′-CTTTATCCCCCACTGACAGC-3′
	Probe	5′-GCTGGAAACCCTGGAAGCCCC-3′
Surfactant protein D	forward	5′-CAGACAGTGCTGCTCTGAGG-3′
	reverse	5′-GCTGTATGGCAGCATTCTCA-3′
	Probe	5′-TGGCCTCCCCACGTTCTGCT-3′

### Mice infection

Groups of six BALB/c and C57Bl/6 mice were infected intranasally with 34 to 46 CFU (colony forming unit) of KHW. For control groups, mice were administrated with 1X PBS. At each time-point (4 h, 24 h, 48 h and 72 h) post infection, one group of KHW-infected and one group of control mice were euthanasized using CO_2_ and the lungs of the mice were homogenized in 1X PBS. For determination of expression levels of the antimicrobial peptide genes, lysozyme, CCL20 and SLPI, the lung homogenate was resuspended in 2 volumes of Trizol LS reagent (Invitrogen, Carlsbad, California) and RNA extraction and real-time quantitative RT-PCR were carried out as described above.

### Statistical analysis

Statistical analysis was done with Student's *t* test. A probability of *P*<0.05 was considered significant. All experiments were conducted at least twice and experimental data were presented as mean value ± the standard deviation of experimental triplicates.

## Results

### *B. pseudomallei* can invade and multiply in murine lung epithelial cells

In order to assess the invasiveness and multiplication of *B. pseudomallei* in LA-4 cells, we quantified the number of intracellular bacteria at 0 h, 2 h, 6 h and 24 h post KHW infection. Upon infection with KHW, 0.114±0.017% of the total inoculated bacteria were internalized into LA-4 cells (Table 2). After entry into the host cells, the bacteria multiplied rapidly and by 24 h post infection, there was a 43.7-fold increase in the number of intracellular bacteria. During these time intervals, more than 80% of the *B. pseudomallei*-infected cells remained viable as determined by measurement of lactate dehydrogenase (LDH) activity released from damaged cells (data not shown).

**Table 2:** Invasion and multiplication of *B. pseudomallei* KHW in LA-4 cells.

Time (Hours) post KHW infection	Number of internalized bacteria (log_10_ of CFU/10^6^ LA-4 cells)^a^	Percentage (%) of internalization b	Fold increase c
0	3.18±1.78	0.114±0.017	N.A
2	3.56±1.87	N.A	2.4
6	4.00±2.11	N.A	6.6
24	4.82±2.06	N.A	43.7

a Number of internalized bacteria after infection with KHW.

b Ratio of internalized bacteria to total inoculated bacteria expressed as a percentage.

c Fold increase in the number of intracellular bacteria compared to the number of intracellular bacteria at 0 h post KHW infection.

N.A- Not applicable

### TLR2 and TLR4 are expressed in murine lung epithelial cells and up-regulated upon *B. pseudomallei* infection

**Figure 2:**
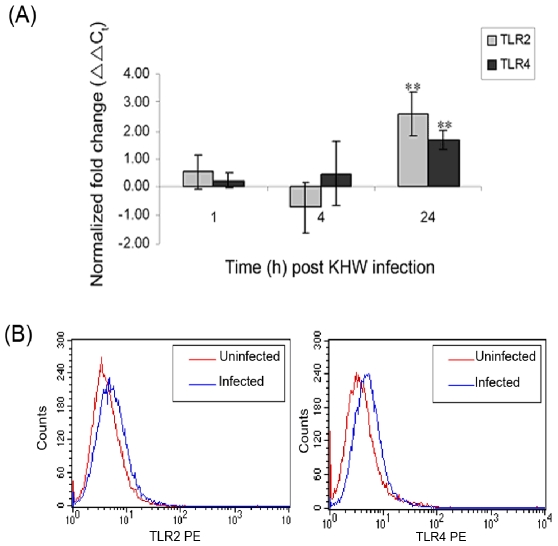
Expression of TLR2 and TLR4 in LA-4 murine lung epithelial cells following *B. pseudomallei* infection using (A) qRT-PCR and (B) flow cytometry. LA-4 cells were infected with KHW for 2 h and control cells were treated with F12K medium. The expression of TLR2 (▪) and TLR4 (▪) genes at 1 h, 4 h and 24 h post infection was quantified by qRT-PCR. Results are normalized to both β-actin and uninfected control cells, and were expressed as normalized fold change (ΔΔC_t_) ± standard deviation of experimental triplicates. ** P<0.05 compared to uninfected control cells (A). Cell surface density of TLR2 and TLR4 was also assessed by staining the cells with anti-mouse TLR2 and TLR4 antibodies prior analysis using FACSArray (B). All results shown are representation of three independent experiments.

To explore the responsiveness of LA-4 murine lung epithelial cells to *B. pseudomallei* infection, we tested for the expression level of TLR2 and TLR4 in the cells using qRT-PCR. TLR2 and TLR4 transcripts were detected in uninfected LA-4 cells (Data not shown). Upon infection with *B. pseudomallei*, the expression levels of TLR2 and TLR4 increased gradually and a significant up-regulation compared to control cells was observed at 24 h post infection (p<0.05) (Figure 2A). Correspondingly, flow cytometry analysis also revealed increased level of cell surface density of TLR2 and TLR4 in *B. pseudomallei*- infected cells at 24 h post infection (Figure 2B).

### *B. pseudomallei* infection of murine lung epithelial cells induces the secretion of proinflammatory mediators

**Figure 3:**
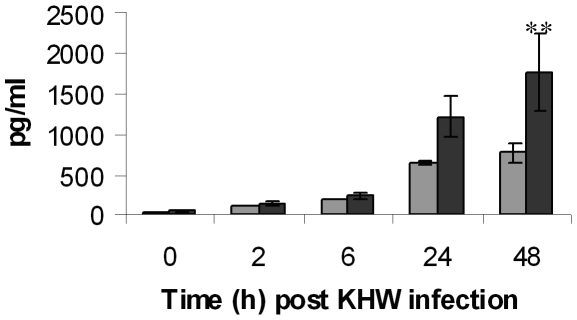
Level of MCP-1 secreted by LA-4 murine lung epithelial cell line following *B. pseudomallei*, KHW infection. LA-4 cells were infected with KHW and control cells were treated with F12K medium. At 0 h, 2 h, 6 h, 24 h and 48 h post infection, supernatants from KHW-infected (▪) and control (▪) cells were harvested and the levels of MCP-1 was measured using FACSArray Bioanalyzer and Cytometric Bead Array. Results are expressed as mean ± standard deviation of the triplicates, and are representation of two independent experiments. ** P<0.05 compared to uninfected control cells.

The release of a panel of cytokines and chemokines including IL-10, IL-12p70, IFNγ, IL-6, MCP-1 and TNFα in LA-4 murine lung epithelial cells was then determined as a measure of inflammatory responses against *B. pseudomallei* infection. Upon infection with *B. pseudomallei*, there was a significant increase in the level of secreted MCP-1 compared to control cells (p<0.05) at 48 h post *B. pseudomallei* infection (Figure 3). However, no significant increase in IL-10, IL-12p70, IFNγ, IL-6, and TNFα was detected (data not shown).

**Figure 4:**
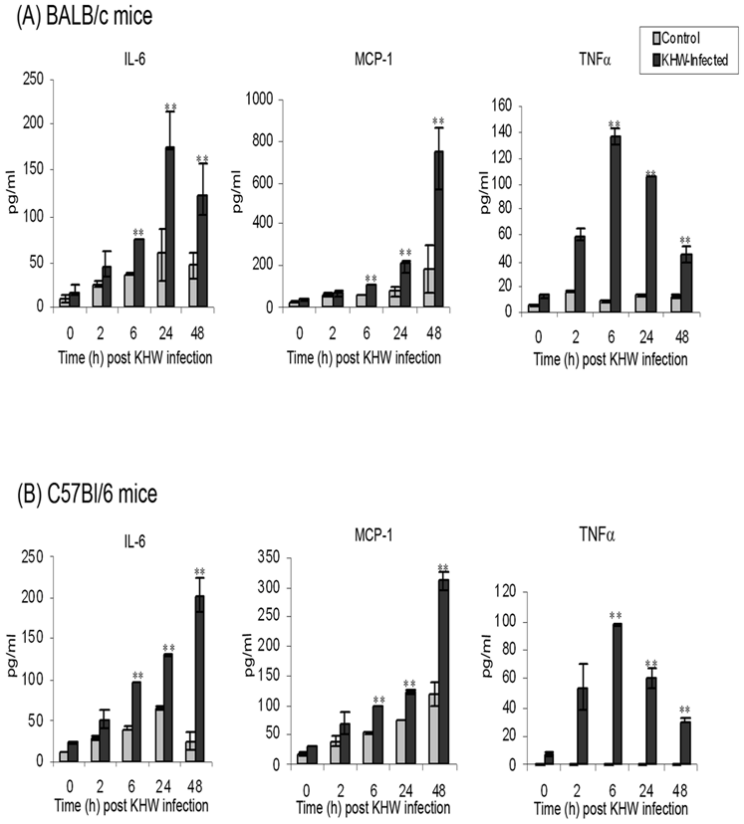
Levels of cytokines and chemokines secreted by primary murine lung epithelial cells from (A) BALB/c mice, and (B) C57Bl/6 mice following *B. pseudomallei*, KHW infection. Primary lung epithelial cells were infected with KHW and control cells were treated with F12K medium. At 0 h, 2 h, 6 h, 24 h and 48 h post infection, supernatants from KHW-infected (▪) and control (▪) cells were harvested and the levels of (A) IL-6, (B) MCP-1 and (C) TNFα were measured using FACSArray Bioanalyzer and Cytometric Bead Array. Results are expressed as mean ± standard deviation of the triplicates, and are representation of three independent experiments. ** P<0.05 compared to uninfected control cells.

This data was further validated using primary epithelial cells isolated from the lungs of BALB/c and C57Bl/6 mice. Infection of primary lung epithelial cells from BALB/c and C57Bl/6 mice with *B. pseudomllei*, KHW resulted in similar infection kinetics (Table S1) and cell monolayer integrity as in LA-4 cells. However, significant increase in the production of IL-6 and TNFα in addition to MCP-1 was observed (p<0.05) (Figure 4). C57Bl/6 primary lung epithelial cells produced an increasing amount of IL-6 in a time-dependent manner in response to KHW infection. On the other hand, BALB/c primary lung epithelial cells produced a time-dependent increase in IL-6 only at 6 h and 24 h after which a decline in the IL-6 production was noted at 48 h post infection. For both the BALB/c and C57Bl/6 primary lung epithelial cells, TNFα production peaked at 6 h post infection and declined gradually at 24 h and 48 h post infection. In addition, the trend of MCP-1 production in these primary cells was different from LA-4 cells. In the primary lung epithelial cells from BALB/c and C57Bl/6 mice, the significant increase in the production of MCP-1 was observed as early as 6 h post infection. A time-dependent induction of MCP-1 production by both BALB/c and C57Bl/6 primary lung epithelial cells was also observed from 6 h to 48 h post KHW infection. These results suggest that cytokines and chemokines are up-regulated when lung epithelial cells are inoculated with *B. pseudomallei*.

### *Burkholderia pseudomallei* infection induces differential expression of antimicrobial peptide genes in murine lung epithelial cells

**Figure 5:**
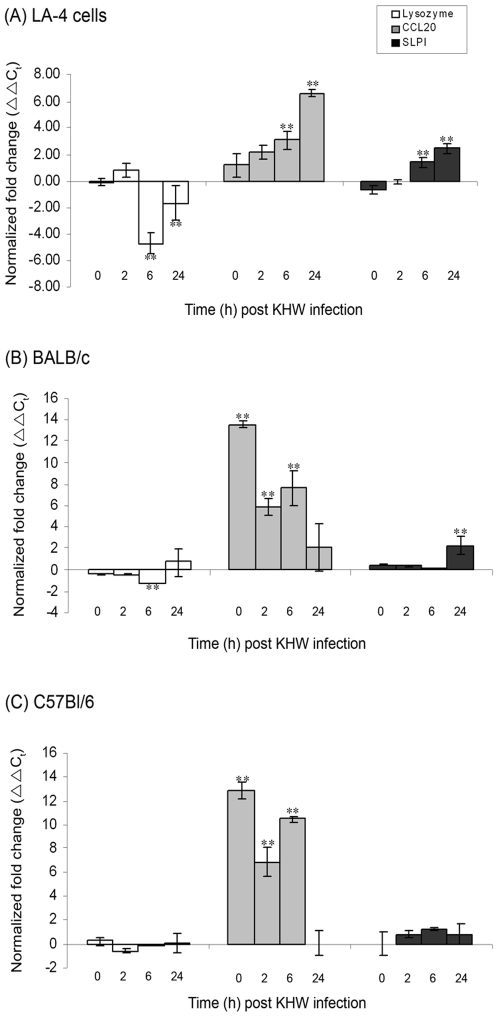
Differential expression of antimicrobial peptide genes in (A) LA-4 murine lung epithelial cell line and primary lung epithelial cells from (B) BALB/c and (C) C57Bl/6 mice following KHW infection. Every 4 bars represent 0 h, 2 h, 6 h and 24 h post infection. Lung epithelial cells were infected with KHW. RNA was extracted from the cells at 0 h, 2 h, 6 h and 24 h post infection and the expression of lysozyme (□), CCL20 (▪) and SLPI (▪) genes was quantified by qRT-PCR. Results are normalized to both β-actin and uninfected control cells, and were expressed as normalized fold change (ΔΔC_t_) ± standard deviation of experimental triplicates. All results shown are representation of three independent experiments. ** P<0.05 compared to uninfected control cells.

To determine the relevance of antimicrobial peptides in epithelial innate immune defence against *B. pseudomallei*, we studied the expression of various antimicrobial peptide genes (murine cathelicidin-related antimicrobial peptide CRAMP, lysozyme, CCL20, SLPI, Surfactant protein A, Surfactant protein D, β-Defensin14) in KHW-infected LA-4 murine lung epithelial cells and primary BALB/c and C57Bl/6 lung epithelial cells using qRT-PCR. We observed differential expression only for lysozyme, CCL20 and SLPI, but not for CRAMP, Surfactant A, Surfactant D and β-Defensin14. In LA-4 cells, infection with *B. pseudomallei* resulted in significant down-regulation of lysozyme and significant up-regulation of CCL20 and SLPI at 6 h and 24 h post infection compared to uninfected control cells (p<0.05) (Figure 5A). In the primary lung epithelial cells from BALB/c and C57Bl/6 mice, significant up-regulation of CCL20 was observed earlier at 0 h, 2 h and 6 h post infection (p<0.05), after which the expression level of CCL20 declined to basal level. However, significant down-regulation of lysozyme was observed only at 6 h for BALB/c mice (p<0.05) while up-regulation of SLPI was observed only at 24 h post infection in BALB/c mice (p<0.05) (Figure 5B and 5C).

### Antimicrobial peptide genes are also differentially regulated in *in vivo* mouse model

**Figure 6:**
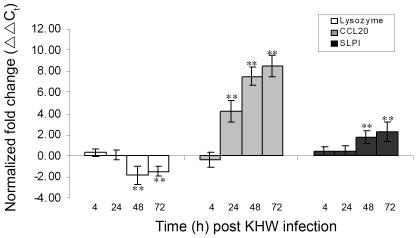
Differential expression of antimicrobial peptide genes in the lung of BALB/c mice following *B. pseudomallei*, KHW infection. Groups of 6 BALB/c mice were treated intranasally with 34 CFU of KHW. Mice in the control group were treated with 1X PBS. At 4 h, 24 h, 48 h and 72 h post KHW infection, RNA was extracted from the lungs of infected and control mice and expression of lysozyme (□), CCL20 (▪) and SLPI (▪) was then determined using qRT-PCR. Results are normalized to both β-actin and uninfected control cells, and were expressed as normalized fold change (ΔΔC_t_) ± standard deviation of six mice. All results shown are representation of two independent experiments. ** P<0.05 compared to uninfected control mice.

To further investigate the significance of these observed differential expression of antimicrobial peptide genes, lysozyme, CCL20 and SLPI, BALB/c mice were infected intranasally with 34 to 46 CFU of KHW, equivalent to twice the lethal dose 50, LD_50_, as determined previously [7]. The expression of the antimicrobial peptide genes, lysozyme, CCL20 and SLPI, at 4 h, 24 h, 48 h and 72 h post infection was determined using qRT-PCR. Only results from one infection experiment (34 CFU) was shown. Upon infection, the bacteria multiplied rapidly in the host and by 72 h post infection, the bacteria load in the lung had increased to 6.79±5.12 log_10_ of CFU of bacteria (Table 3). Correspondingly, CCL20 was significantly up-regulated at 24 h, 48 h and 72 h post infection (p<0.05) and SLPI was significantly up-regulated at 48 h and 72 h post infection (p<0.05) (Figure 6). Conversely, significant down-regulation of lysozyme gene was also observed at 48 h and 72 h post infection (p<0.05). The general agreement of these *in vivo* findings with the primary lung epithelial cells infection model with *B. pseudomallei* indicates that epithelial cells play a significant role in antimicrobial response against pulmonary *B. pseudomallei* infection.

**Table 3:** Bacterial load in lung of KHW-infected mice at 0 h, 4 h, 24 h, 48 h and 72 h post infection.

Time (Hours) post KHW infection	Bacterial load in the lung (log_10_ of CFU)
0	0.87±0.4
4	1.36±1.15
24	3.13±2.75
48	5.83±4.81
72	6.79±5.12

### Lysozyme, SLPI and CCL20 are regulated by the NF-κB and p38 MAPK pathways

**Figure 7:**
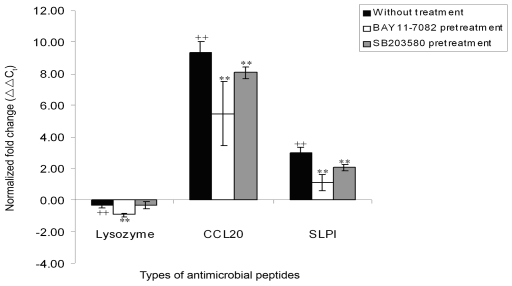
Regulation of lysozyme, CCL20 and SLPI by NF-κB and p38 MAPK pathways. LA-4 cells were pre-incubated with or without BAY11-7082 (10 µM) and SB203580 (10 µM) for 1 h prior to KHW infection; without treatment (▪), BAY11-7082 pretreatment (□), SB203580 pretreatment (▪). Total RNA was extracted from the cells at 24 h post KHW infection and the expression of lysozyme, CCL20 and SLPI was determined using qRT-PCR. Results are normalized to both β-actin and uninfected control cells, and were expressed as normalized fold change (ΔΔC_t_) ± standard deviation of experimental triplicates. All results shown are representation of three independent experiments. ^++^P<0.05 compared to uninfected control cells, ^**^P<0.05 compared to untreated control cells.

In order to elucidate the pathways that are involved in the regulated expression of the antimicrobial peptide genes, lysozyme, CCL20 and SLPI, LA-4 cells were pre-incubated with specific inhibitors of NF-κB and p38 MAPK pathways (BAY11-7082 and SB203580 respectively) prior to the infection process. Pre-incubation of the epithelial cells with BAY11-7082 (10 µM) significantly impaired the up-regulation of all three CCL20, lysozyme and SLPI genes upon *B. pseudomallei* KHW infection compared to untreated cell controls (p<0.05) (Figure 7). On the other hand, pre-treatment with SB203580 (10 µM) resulted in significant reduction in the up-regulation of only CCL20 and SLPI genes upon *B. pseudomallei* KHW infection compared to controls (p<0.05). Similar inhibitory trends were observed for all 3 different concentrations (5 µM, 10 µM and 25 µM) of SB203580 and BAY11-7082 (Figure S1).

## Discussion

Host innate immune response is a crucial event for early containment of *B. pseudomallei* infection due to the long latency period between pathogen exposure to the onset of clinical symptoms and the time taken for diagnosis of the disease due to its wide range of clinical manifestations [22], [23]. Currently, in melioidosis, studies on the initial innate immune response to *B. pseudomallei* infection have focused on macrophages, neutrophils and NK cells [24], [25]. Epithelial cells occupy a large surface area in the lung and could play a central role in inducing protective immune response against microbial pathogens [26]–[32]. Pulmonary epithelial cells could also represent one of the most immediate targets in a pulmonary *B. pseudomallei* infection. However, knowledge on how epithelial cells respond to *B. pseudomallei* is limited. In the current study, using a well-established murine lung epithelial cell line, LA-4, and primary lung epithelial cells isolated from the lungs of BALB/c and C57Bl/6 mice, we sought to characterize for the first time the immune factors that are involved in epithelial-mediated immunoreaction against *B. pseudomallei* infection in the lung of infected host. We demonstrated that LA-4 mouse lung epithelial cells express TLR2 and TLR4, the innate immunity receptors in Gram-negative bacteria, and these receptors were up-regulated upon exposure to *B. pseudomallei*. TLR activation initiates the expression of important mediators and was crucial for inducing the innate immune responses in the epithelium and causing subsequent inflammation [31].

Cytokine secretion is an early event in the innate host response that alerts the immune system to the presence of a microbial pathogen. Infection of human alveolar epithelial cells with *Francisella tularensis* resulted in increase levels of multiple cytokines including granulocyte-macrophage colony-stimulating factor (GM-CSF), IL-8, MCP-1 and macrophage inflammatory protein 1α (MIP-1α) [33]. Induction of IL-6 gene expression was also observed in bronchial epithelial cells infected with *Pseudomonas aeruginosa* [34]. In *B. pseudomallei-*infected murine lung epithelial cells, the levels of IL-6, MCP-1 and TNFα were increased significantly compared to the uninfected control cells. In addition, substantial increase in cytokine production was observed much earlier in the primary cells (6 h) as opposed to the cell line (24 h), possibly due to altered immune responses in the LA-4 lung adenoma cells. The kinetics of cytokine production by these murine lung epithelial cells differs slightly from those previously reported in the lungs of *B. pseudomallei-*infected mice [7]; specifically IFNγ was not detected in the epithelial cells. This discrepancy is likely to be due to the fact that NK cells are the main sources of IFNγ in the lung [23], and that epithelial cells are incapable of IFNγ production.

The production of IL-6, TNFα and MCP-1 by murine lung epithelial cells, however, were consistent with previously reported in vivo studies [7], [35]. In pneumonic melioidosis patients, elevated plasma levels of IL-6 and TNFα have also been described [36], [37]. IL-6 and TNFα were previously known to play an important role in controlling the rapid dissemination of the bacteria upon infection [23], [35]. Although Easton *et. al.* (2007) have shown that neutrophils are the major producer of IL-6 and TNFα in the lungs [24], our data extends this observation to epithelial cells, which also appear to be an important source of IL-6 and TNFα. In fact, epithelial IL-6 has also been suggested to increase neutrophil intracellular calcium ions, causing neutrophil activation [38]. Besides IL-6 and TNFα, epithelial cells also appear to be another important source of MCP-1 production in the lungs. MCP-1 is a protein that attracts and activates mononuclear cells and may be a critical regulator of mononuclear cell migration [39]. In *Listeria monocytogenes*, it was determined using a murine model that MCP-1 was an important chemotactic factor for mononuclear cells [40]. Clearance of the bacteria was observed upon the recruitment of monocytes. Deletion of MCP-1 gene resulted in partially impaired monocyte recruitment, reduced clearance of bacteria in the spleen and enhanced susceptibility to infection [40]. Thus, our findings that murine lung epithelial cells were able to secrete IL-6, TNFα and MCP-1 suggest that lung epithelial cells are capable of early production of pro-inflammatory cytokines and chemokines, which are important components in the innate immune response.

It is known that endogenously produced antimicrobial proteins are associated with host defense against bacterial infections [15], [41], [42]. However, the role of antimicrobial peptide in pulmonary infection by *B. pseudomallei* has not been well-studied. Our current study addresses the expression profile of a wide spectrum of antimicrobial peptides (CRAMP, lysozyme, CCL20, SLPI, Surfactant A, Surfactant D, β-Defensin14) in murine lung epithelial cells upon *B. pseudomallei* infection. Our findings showing up-regulation of CCL20 and SLPI in *B. pseudomallei*-infected *in vitro* cultured cells and *in vivo* mouse model indicate an epithelial-initiated anti-microbial response against *B. pseudomallei*. Indeed, previous studies have shown that the up-regulation of CCL20 followed by its interaction with CC chemokine receptor 6 (CCR6) is responsible for the chemoattraction of immature dendritic cells (DC), effector/memory T-cells and B-cells to the site of inflammation [43], [44]. CCL20 has been shown to exert antimicrobial activity against a wide spectrum of Gram-negative bacteria [45]. SLPI was also known to exhibit multiple functions, including inhibition of protease activity, microbial growth, and inflammatory responses [46]. In *Streptococcus pneumoniae*-infected mice, the amount of SLPI mRNA transcripts in the lung was three times higher than the baseline at 10 h after bacterial inoculation [47]. Interestingly, we observed significant down-regulation in the lysozyme gene expression in the epithelial cells upon *B. pseudomallei* infection. An increased expression of lysozyme has been shown to be associated with enhanced bacterial killing, decreased systemic dissemination and improved survival following infection [48]. The observation that lysozyme gene was down-regulated during *B. pseudomallei* infection could thus imply that lysozyme is dispensable against *B. pseudomallei* infection. Alternatively, it suggests that *B. pseudomallei* may have an immune-escape mechanism to suppress its expression in order to promote its survivability in the host. Experiment to investigate the role of lysozyme in *B. pseudomallei* colonization and dissemination is currently underway. Similar down-regulation of genes encoding antimicrobial peptides have previously been detected in gut mucosa biopsies from patients with shigellosis and in *Shigella*-infected epithelial cells [49], [50]. Nevertheless, our findings that CCL20 and SLPI genes were up-regulated and lysozyme gene was down-regulated following *B. pseudomallei* infection indicate that *B. pseudomallei* could interfere with host regulation of genes encoding different antimicrobial peptides in different ways.

Activation of cell signaling pathways is crucial for the induction of antimicrobial genes expression upon pathogen exposure. In gastric epithelial cells, infection with *Helicobacter pylori* results in the activation of the NF-kB pathway, leading to CCL20 gene transcription [51]. Furthermore, blockage of the NF-kB and p38 MAPK pathways impaired CCL20 production [52]. We demonstrated in pulmonary epithelial cells that antimicrobial peptides CCL20 and SLPI were regulated by both NF-κB pathway and p38 MAPK pathway, whereas lysozyme was regulated only by NF-κB pathway. Our results therefore suggest that cell signaling pathways, NF-κB as well as p38 MAPK, govern the expression of specific antimicrobial peptides in pulmonary epithelial cells.

The findings of our study indicate that pulmonary epithelial cells play important roles in the innate immune response against *B. pseudomallei* in the lung and cannot be ignored in studies investigating the pathogenesis of pulmonary melioidosis. Further studies on whether the immune factors identified here confer protection against melioidosis could lead to the identification of important therapeutic targets.

## Supporting Information

Figure S1 (0.25 MB DOC)

Table S1 (0.03 MB DOC)
