# Supplementary material for: Innate Immune Responses of Pulmonary Epithelial Cells to Burkholderia pseudomallei Infection
Source: PLoS One. 2009 Oct 6;4(10):e7308. doi: 10.1371/journal.pone.0007308 (PMC2751829; doi:10.1371/journal.pone.0007308)
Supplement: Figure S1 — (0.25 MB DOC) [file pone.0007308.s001.doc]

**Figure S1**

Results showed that pre-incubation of epithelial cells with all 3 different concentrations of BAY11-7082 (5µM, 10µM and 25µM) prior to *B. pseudomallei* infection significantly impaired the up-regulation of lysozyme, SLPI and CCL20 compared to the untreated control cells and this inhibitory effect was not dose-dependent. On the other hand, pre-incubation of epithelial cells with all 3 different concentrations of SB203580 (5µM, 10µM and 25µM) prior to *B. pseudomallei* infection also resulted in dose-independent reduction of the expression of SLPI and CCL20.

10µM of BAY11-7082 and SB203580 was finally chosen because this concentration of inhibitor was utilized in studies by other researchers on epithelial cell signaling (Zhao *et al*, 2008; Zhang *et al*, 2007; Méndez-Samperio *et al*, 2009).


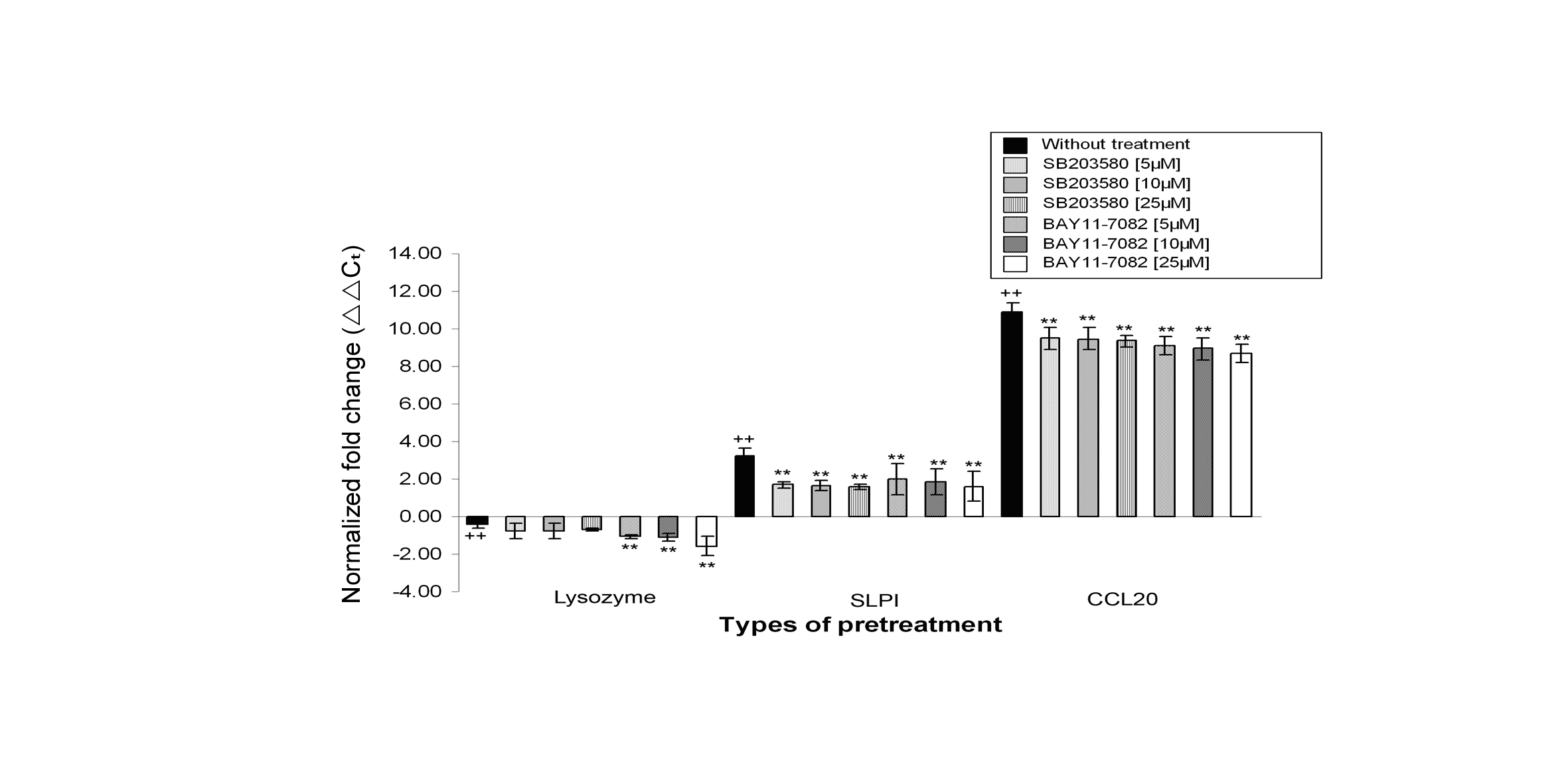


**Effects of p38 MAPK and NF-κB pathway inhibitors on *B. pseudomallei*-regulated lysozyme, SLPI and CCL20 expression.** LA-4 cells were pre-incubated with or without SB203580 (5, 10 and 25 µM) and BAY11-7082 (5, 10 and 25 µM) for 1 h prior to *B. pseudomallei* KHW infection. Total RNA was extracted from the cells at 24 h post KHW infection and the expression of lysozyme, CCL20 and SLPI was determined using qRT-PCR. Results are normalized to both β-actin and uninfected control cells, and were expressed as normalized fold change (ΔΔC_t_) ± standard deviation of experimental triplicates. All results shown were representation of three independent experiments. ^++^P < 0.05 compared to uninfected control cells, ^**^P < 0.05 compared to untreated control cells.

**References**

Zhao YT, Guo JH, Wu ZL, Xiong Y, Zhou WL. (2008) Innate immune responses of epididymal epithelial cells to Staphylococcus aureus infection. Immunol Lett 119: 84-90.

Zhang Z, Reenstra W, Weiner DJ, Louboutin JP, Wilson JM. (2007) The p38 mitogen-activated protein kinase signaling pathway is coupled to Toll-like receptor 5 to mediate gene regulation in response to Pseudomonas aeruginosa infection in human airway epithelial cells. Infect Immun 75: 5985-92.

Méndez-Samperio P, Pérez A, Rivera L. (2009) Mycobacterium bovis Bacillus Calmette-Guérin (BCG)-induced activation of PI3K/Akt and NF-kB signaling pathways regulates expression of CXCL10 in epithelial cells. Cell Immunol 256: 12-8.
