# Supplementary material for: Innate Immune Responses of Pulmonary Epithelial Cells to Burkholderia pseudomallei Infection
Source: PLoS One. 2009 Oct 6;4(10):e7308. doi: 10.1371/journal.pone.0007308 (PMC2751829; doi:10.1371/journal.pone.0007308)
Supplement: Table S1 — (0.03 MB DOC) [file pone.0007308.s002.doc]

**Table S1**

Infection of primary lung epithelial cells from BALB/c and C57Bl/6 mice with KHW resulted in internalization of 0.116±0.045% and 0.127±0.034% of the total inoculated bacteria into the host cells respectively. As in LA-4 cells, the bacteria multiplied rapidly in the host cells and by 24 h post infection, there was a 44.7-fold and 55.0-fold increase in the number of intracellular bacteria in the primary lung epithelial cells from BALB/c and C57Bl/6 mice.

**Invasion and multiplication of *B. pseudomallei* KHW in primary lung epithelial cells from BALB/c and C57Bl/6 mice**

| Time (Hours) post KHW infection | BALB/c primary lung epithelial cells | | | C57Bl/6 primary lung epithelial cells | | |
| --- | --- | --- | --- | --- | --- | --- |
|  | Number of internalized bacteria (log_10_ of CFU/10^6^ cells)^a^ | Percentage (%) of internalization ^b^ | Fold increase ^c^ | Number of internalized bacteria (log_10_ of CFU/10^6^ cells)^a^ | Percentage (%) of internalization ^b^ | Fold increase ^c^ |
| 0 | 3.11±0.91 | 0.116±0.045 | N.A | 3.15±1.51 | 0.127±0.034 | N.A |
| 2 | 3.43±1.38 | N.A | 2.09 | 3.54±1.81 | N.A | 2.45 |
| 6 | 3.79±1.92 | N.A | 4.79 | 3.83±2.24 | N.A | 4.78 |
| 24 | 4.76±3.01 | N.A | 44.7 | 4.89±3.02 | N.A | 55.0 |
